# The Importance of the Eugenic Movement and Its Relation to Social Hygiene*Extracts from a paper read before the Maryland Society of Social Hygiene, April 19, 1910.

**Published:** 1913-10

**Authors:** Lewellys F. Barker

**Affiliations:** Professor of Medicine in Johns Hopkins University and Physician-in-Chief to Johns Hopkins Hospital, Baltimore


### Department of Eugenics.

Conducted by Dr. Malone Duggan and Dr. Theo. Y. Hull.

### THE TEXAS STATE SOCIETY OF SOCIAL HYGIENE.

Headquarters: San Antonio, Texas.

Dr. Malone Duggan, President, San Antonio.
Dr. F. E. Daniel, 1st Vice President, Austin.
Prof. A. Caswell Ellis, 2d Vice President, Austin.
Mrs. Eli Hertzberg, 3d Vice President, San Antonio.
Dr. Theo. Y. Hull, Secretary, San Antonio.
Mr. W. L. Evans, Treasurer, San Antonio.

EXECUTIVE COMMITTEE.

Dr. Malone Duggan, Chm. | Hon. J. C. Willacy. | Dr. W. A. King.

Dr. Theo. Y. Hull, Sec'y. | Mr. W. L. Evans. | Dr. J. W. Torbett.

Dr. W. F. McCaleb. | Dr. Frank D. Boyd. | Dr. F. C. Walsh.

Hon. C. H. Jenkins. | Dr. J. R. Nichols. | Dr. Joe Reuss.

Rev. A. W. S. Garden. | Dr. J. M. McCutchan. | Dr. Frank Paschal.

### The Importance of the Eugenic Movement and its Relation to Social Hygiene.*

BY LEWELLYS F. BARKER, M. D., PROFESSOR OF MEDICINE IN JOHNS HOPKINS UNIVERSITY AND PHYSICIAN-IN-CHIEF TO JOHNS HOPKINS HOSPITAL, BALTIMORE.

"Like leaves on trees the race of man is found,
Now green in youth, now withering on the ground;
Another race the following spring supplies
They fall successive and successive rise."

If it be true that the whole human race gets a fresh start at least thrice every hundred years; that the kind of race in existence in any period depends on the quality of the children born and the influences to which they the exposed after birth; that the supreme reason for marriage is parenthood; that the quality of children born depends (1) on the elements inherent in the germ-cells of the parents, and (2) on the environment of those germ-cells before their union and also after their fusion to form a new being; that a critical study of the relatives of a prospective parent will give valuable evidence regarding the nature of the elements—good, bad and indifferent—inherent in his or her germ-cells and likely to be transmitted; that poisons like alcohol, lead, or the toxins of syphilis circulating in the blood of either parent before a germ-cell

*Extracts from a paper read before the Maryland Society of Social Hygiene, April 19, 1910.

union, or, and more especially, in the blood of a mother before the birth of her child, may make the offspring the victim of malformation, infantilism, mental defect or epilepsy—if these things be true I think it probable that it would be possible and wise to interest young and marriageable people in the facts, and to a certain extent consciously to direct selection by marriage, and further, consciously to protect from injury germ-cells which possibly may fuse, or have already united to form a new human being.

### CREATE PUBLIC OPINION.

If it be true that the quality of human beings born depends on the inherent qualities of the germ-cells of the parents and their environment before and after fusion, may it not be possible by education to cultivate a public opinion that will lead families to show as much pride in making provision for characteristics of health, energy, ability, courage and courtesy in their posterity as some of them now evince in their ancestry? May not family sentiment be developed in the direction of suppressing parenthood in the racially unworthy and of facilitating fatherhood and motherhood for those who are able to transmit to their offspring desirable qualities unhampered by glaring defect? In the words of Zarathustra, may we not say to the family: "In the future let not your pride be whence ye have come, but whither ye go."

If it be true that those born deaf and dumb, or feeble-minded, or with an insane or epileptic tendency, or color-blind, are likely to propagate their kind; and if it be true that the chronic inebriate, the prostitute, the drug habitue and the habitual criminal are, as a rule, such, because of the nervous systems they have inherited, and that they are likely to transmit defective nervous systems to their offspring, it would be desirable to educate public opinion, not only to discountenancing, but actually to legislative prohibition of parenthood for individuals so afflicted.

### THE FIT AND THE UNFIT.

If it be true that the decline and fall of nations following from no apparent external causes is really explicable through "the relative fertility of the fitter and unfitter elements combined with what we now know of the laws of inheritance;" if it be a fact that the success of a people usually leads to a cessation of natural selection within that people, and hence is often followed by survival of the worse; if we can be convinced that by applying scientific knowledge it would be possible to stem racial decadence and determine a racial ascent, surely there could be no more laudable, patriotic movement than one which will help a nation in its struggle by seeing to it that the children of that nation have their origin in the fusion of the better germ-cells available, and that those germ-cells and those children are protected from the injury which results from under-nourishment or from the action of such racial poisons as alcohol, lead, and the toxins produced by infectious agents. We do not have to look far afield to observe that certain of the civilizations in existence today are trying to apply every scrap of biologic knowledge which they believe will help them in the struggle; woe be to the societies and nations which fail to apply this new knowledge as it accumulates; the fate of such negligent or non-compliant communities is sealed; they are making no proper effort to postpone the time when they will be "one with Nineveh and Tyre."

If it be true that as the human race advances and the range of intellect widens, the tendency is to work for the future of humanity as well as for its present good; if, from the time of Plato to our day, the best minds have cherished the idea of a more perfect state, and have urged the suppression of human baseness and the creation and the appreciation of a "super-man;" if modern science has placed in our hands the key which unlocks the box containing the secret of race-ennoblement; namely, the picking out by preference of the racially superior for parenthood combined with the protection of childhood and the support of maternal care by fatherhood—if these be facts, then the phenomena of sex may ultimately come to be regarded by more people with awe; parenthood will be looked on as the noblest and most sacred of functions, entailing the heavist responsibilities, and "the science which deals with all the influences that improve the inborn qualities of a race" (Mr. Francis Galton's definition of eugenics), will become a constituent of the higher human religions.

### SOME PRINCIPLES IN HEREDITY.

For a long time it has been known that most animals and plants begin life by the union of two cells, the one male, the other female. Biologists describing those cells call them gametes; that is, germ-cells or marrying-cells. These germ-cells contain in some way or another the factors on which all the powers, both physical and mental, possessed by the living creature to which they give rise, depend. It was Mendel who first appreciated the full consequences of the double nature of each individual being, due to the fact that two cells are concerned in its production. To understand the facts of heredity it will no longer do to think of a boy or a bee or a beech-tree as each being one thing, but we must think of each of them as being two things, indeed, as being double in composition throughout.

It is no uncommon experience to see the attempt made in families to analyze each individual into the aggregations or mixtures of parental characteristics manifest in them, to recognize, for example, the mother's voice, the father's passion for music, the mother's eye or the father's bony frame-work. But scientific students of heredity teach us that such an analysis, while true, since the various characters are transmitted independently, misses the most important point; namely, that in each of his qualities an individual is double. To understand really the make-up of an individual it is necessary to know how each of the two original germ-cells which united to give rise to him was endowed, as regards the factors concerned in voice, skeleton, musical faculty, color, hair and other characteristics. In analyzing the inheritance of a human being, therefore, we have to consider the contribution of each of his parental germ-cells separately for each quality or faculty; in other words, it is not sufficient to make a single column of values for the ingredients that go to form a man, but, as Bateson suggests, we must rule two columns, one for the ovum from the mother and one for the sperm-cell from the father, and in each column we must indicate how that germ-cell was supplied in respect to each of the ingredients in the list. Now, it will be clear that the male and female germ-cells may make the same or different contributions as regards any of the ingredients. When the two cells yield the same contribution the organism which results is said to be *pure-bred* for that ingredient; on the other hand, when the contribution from the two parent cells is dissimilar, the organism which results is said to be *cross-bred*. This is one of the most important conclusions to which studies of heredity has led.

From what I have said it will be clear that an individual according to our present views of heredity, is made up of a large number of distinct ingredients or units contributed from two sources and that as regards any one of these units he may have inherited two similar portions or two dissimilar portions.

To proceed with the analysis, I think I cannot do better than adopt an illustration used by Bateson in his popular address. He imagines the contents of a germ-cell as a fluid, made by taking a drop from each of a definite number of bottles in a chest containing tinctures of the several ingredients. Thus the male germ-cell is supposed to be compounded from one chest of bottles and the female germ-cell from a corresponding set of bottles in a similar chest. Should one or more of the bottles be empty in either chest then the germ-cell receives no ingredient from that chest; if corresponding bottles should be empty in both chests then neither germ-cell contains the ingredient concerned and the new individual resulting from the union of the two germ-cells by mixing the two collections of drops together would not contain the missing ingredient at all. It is obvious that an individual may be purebred (that is to say, similar on both sides of his composition) for any particular ingredient in either one of two ways; he may have received the ingredient from both the male and the female chest, or he may have received it from neither. Again, he may be crossbred in respect of any single ingredient or unit, receiving the presence of it from one germ-cell and the absence of it from the other.

Having got this far, we can now conceive of the individual as composed of what are called presences and absences of all the possible ingredients. Hurst's studies of the eyes of parents and children happily illustrate this point. It appears that people have blue eyes when a factor or unit which forms pigment on the front of the iris is absent. If both parents of a child have blue eyes the children will not have dark eyes, for a dark eye has been shown to be due to either a single or a double dose of the unit missing from the blue eye. This is why dark-eyed persons may have children who are all dark-eyed or who are dark-eyed and light-eyed in certain proportions which, on the average, are definite.

PRINCIPLE OF SEGREGATION OF UNITS.

Having described the formation of an individual from units brought into him by the two germ-cells derived from his parents, we may now turn to an examination of the mode of formation of the germ-cells which are formed in him himself and which will be concerned in his contribution to his children should he have them. It will be interesting to see how the various ingredients of which he himself was originally compounded are distributed among the germ-cells which are budded off from him. The observations which have been made teach that except for rare variations the germ-cells formed from him are all alike in respect of those units of which he is pure-bred. This might have perhaps been expected, but the constitution of his germ-cells in respect of the units of which he is cross-bred—I mean those units which he received from the germ-cell of one parent and not from that of the other—could scarcely have been foreseen. In the cases thus far studied experimentally in animals and plants some of the germ-cells from such individuals contain a representation of the unit and others do not, just as the parent germ-cells did or did not contain it. This fact, known as the priciple of segregation of units (or determiners), is the most important discovery made by Mendel.

We now see that if a certain unit were brought in by both parent germ-cells, then all the daughter germ-cells have it; if it did not come in through either parent germ-cell, then no daughter germ-cell will have it; if it came in through one parent germ-cell and not through the other, then about half the daughter germ-cells will have it and about half of them will not. In other words, the individual made by the mixing of tinctures from two chests of bottles sorts the tinctures back again into a double row of bottles, a pair corresponding to each ingredient or unit, and each of the germ-cells budded off from him receives a drop from one or other bottle of each pair. This is what is known as the purity of germ-cells. "They are pure in the possession of an ingredient or in not possessing it; and the ingredients or factors, as we generally call them, are units because they are so treated in the process of formation of the new gametes and because they come out of the process of segregation in the same condition as they went in before fertilization." (Bateson.)

Two given parents can therefore produce only a limited number of types of offspring; the relative numbers of the types thus produced by recombinations of the parental units can often be predicted according to simple mathematical rules.

If we could know the unit characteristics which both parents possess as well as those which they both lack, and also the unit characters in which they differ, and if we further knew for each characteristic whether it was due to a presence or an absence, we could go far toward predicting the result of a particular marriage. Of course we are as yet only on the threshold of such knowledge. It is often very difficult to tell whether a parent possesses a given factor or not, but for some characters it is quite easy and can be told at once, since there are factors which cannot be present in an individual without manifesting their presence. In some families, for example, a certain number of the members are affected by night-blindness. If an individual affected marries one unaffected, their children will be partly affected and partly unaffected, but their unaffected children in turn, not containing the unit responsible for night-blindness, cannot pass it on.

RESISTANCE TO DISEASE.

While studies of heredity show us the direction in which we must work on the negative side, they are also full of promise on the positive side. One of the most fascinating investigations with which I am familiar is that of Biffen which led to the demonstration that the power to resist a disease may depend on the presence in a germ-cell of one of the simple Mendelian units to which I have been referring. It has long been known that certain kinds of wheat are very susceptible to a disease known as wheat-rust which is due to an infection with a fungus. Certain other varieties of wheat are immune to this rust disease. If an immune strain be crossed with one which is not immune, the resulting hybrids are all susceptible to rust, but when such hybrids are permitted to undergo self-fertilization they yield seeds from which appear susceptible and immune plants in the ratio of 3:1. This little experiment gives an entirely new and precise significance to what we have hitherto designated as resistance to disease; it is conceivable that a field of research has here been opened up which may yield results of great practical as well as theoretical value. It is quite conceivable that what has been found to be true in this case may turn out to be true of resistance to many other diseases in both plants and animals; and who knows but that before long the attention of medical science will be turned less than at present towards modes of infection and more than now toward the breeding of a race with heightened resistance? Indeed it seems now far from being impossible "that liability to a disease or the power of resisting its attack, addiction to a particular vice or to superstition," may be found to be "due to the presence or absence of a specific ingredient and that these ingredients are transmitted to the offspring according to definite predicable rules."

APPLICATION OF EUGENICS TO RACE CULTURE.

The science of eugenics aims to make use of all these newer facts of heredity as well as those resulting from statistical or biometric inquiries, and to apply them in race culture. It will not, however, neglect the very important influence of environment, and must pay especial attention to the role of racial poisons like alcohol, lead and the toxins of syphilis. Time will not permit me to dwell on the obstacles in the way of eugenics progress, nor can I even refer to all the methods proposed by those who harbor the eugenic ideal. It seems to me important, however, to point out that certain of the methods recommended by enthusiasts for the improvement of the race would, if resorted to, be likely to be more harmful than useful. It seems to me probable that the new facts of heredity may be applied best through the utilization of the institution of marriage. I do not think it probable that any method incompatible with marriage, with the love of the sexes, or with the care of the children chiefly by their own parents, will be adopted, at any rate in the near future. The suggestions of Plato, of Malthus, of Spencer and of Nietzsche, though important in certain directions, were all fallacious in others. It is highly probable that some of the views most generally held today will not bear the test of time, but, despite this, we have enough certain knowledge to permit us to do much toward the elevation of mankind by eugenic methods. Though the birth-rate is decreasing, the population of the world is increasing. Though one-third of the infants born die within five years, great strides are being made in the prevention of infant mortality. It is not necessary to foster infant mortality in the hope of removing the unfit. It is much more important to prevent the birth of the unfit. By understanding Nature and obeying her we shall learn to command her. Most of the illegitimate children now born die soon after birth. The majority of undesirable citizens do not propagate their kind, even under our present social arrangements. Selection by marriage, faulty as it is, has on the whole favored the improvement of the race. Women are becoming freer to chose husbands than ever before and social opinion regarding the length of time of engagements and the prevention of unfit matings is becoming ever more enlightened. It might be advantageous if women especially suited for motherhood were made financially independent by the State. So-called codes of honor found to be wrong are being abandoned. More and more provision is being made for the permanent detention of inebriates, insane, the feeble-minded and criminals; parenthood will be more and more prevented among these degenerate individuals, by permanent detention, or possibly, as has been seriously recommended and practiced by some, by surgical or $x$-ray sterilization. Young people are being educated better than before as to the significance of sex and the responsibility of parenthood, of the dangers of racial poisons, and especially of the risks of venereal disease. With the help of the Wassermann reaction it is now possible to find out whether an individual who has once had syphilis has been sufficiently treated to prevent his transmission of the disease to a conjugal partner or a child.

---

"Ah say, Miz Mandy, am yo' program full?"

"Lordee, no, Mr. Lumley. It takes mo' an a san'wich an' two olives to fill mah program."—*The Coyote.*

